# Retrospect of the Medical Sciences

**Published:** 1843-05-27

**Authors:** 


### ANEURYSM OF ABDOMINAL AORTA OPENING INTO THE LUNGS.

At a meeting of the Pathological Society of Dublin, January 8, 1842, Dr. Stokes said the specimens he then produced to the society were of great pathological interest; they belonged to a case of aneurysm of the abdominal aorta at the commencement of its course, immediately under or between the crura of the diaphragm, and exhibiting a mode of termination that had not as yet been described. In some cases of this affection the opening has been into the pleura; in others, into the stomach, into the peritoneum, or behind the peritoneum, forming tumors. In the case now communicated by Dr. Stokes, the aneurysm had opened into the substance of the lung; the subject of the case had been first seen by Mr. Pakenham, and subsequently by Mr. Colles and Dr. Stokes. He complained of intolerable pain in the lumbar region, which was aggravated by motion, but relieved by lying with the back towards the fire, or by turning on the face. There was strong pulsation in the epigastrium, and a strong bruit de soufflet or bellows murmur. From all the symptoms, Mr. Pakenham considered the case to be one of aneurysm of the abdominal aorta, in consequence of which the consultation was determined on. Dr. Stokes ascertained that the patient had been complaining for eighteen months, and that he suffered pain of two distinct kinds, one of which was dull and was constantly felt; the other was a violent lancinating pain, which only occurred in paroxysms, often darted downwards into the scrotum, and was not observed for the first time until three months after the other had commenced. The seat of pain was along six vertebræ, the three last dorsal, and the three contiguous lumbar; the pain was increased by pressure on any part of that tract, or by any motion of the spine; a little to the left of the epigastrium was the tumor, in which could be felt a pulsation, evidently coincident with the diastole of the heart; when deep pressure was made there was an extreme diastolic force perceptible; the bruit de soufflet was increased by motion, and was not audible posteriorly; the pulse was regular, about 80 in the minute; the appetite bad; towards the termination of the case there was pain in the right shoulder. The patient died on this day (January 8) in a state between syncope and asphyxia, after expectorating a large quantity of blood. On the preceding day his appearance was very haggard, the tumor was pulsating violently, and, contrary to what is usual in these cases, the pulse was singularly rapid.

When the body was opened, a small quantity of blood was found in the stomach, which had probably been swallowed in the last agonies of life; there was no effusion into any of the serous cavities. The lung was prodigiously gorged with blood, much of which had been poured into the bronchial tubes; the surface of the lung was abraded when it was in contact with the aneurysm. The aneurysm itself was not very large; it was situated immediately between the crura of the diaphragm, between which it passed upwards until it came into contact with the inferior lobe of the lung; the aperture in the artery was in its anterior wall. There was no perforation of the diaphragm by absorption, but the tumor had forced its way upwards between the crura, merely dissecting through cellular connections.

Dr. Stokes concluded by remarking, that there were three circumstances in this case which rendered it of great value; first, the diagnosis of the aneurysm from symptoms; second, the confirmation of the stethoscopic signs; third, the peculiar mode of termination, which, as he stated at the commencement, had not been previously described by any pathologist.—*Provincial Medical Journal.* March 11, 1843.

---

### EXPERIMENTS ON THE TORPEDO.

M. Matteucci communicated the results of some new experiments on the torpedo, illustrative of the theory entertained by himself and M. de Blainville on the analogy between muscular contraction and electricity. He introduced a small quantity of the aqueous solution of opium into the stomach of the living torpedo; the tincture of nux vomica was likewise introduced into the stomach of another live torpedo. The two fishes, apparently dead, were soon afterwards removed from the water, and on their backs were placed two frogs (prepared in the way already described by the author,) and the galvanometer. When the animal, or any part of it, was slightly touched, it contracted, and the torpedo furnished an electrical discharge, although before the experiment it required strong irritation to produce any effect.

The brain of a torpedo, much reduced in strength, was exposed, and an alkaline solution of potash applied on the fourth lobe. The torpedo died, giving forth very strong discharges.

The electrical organ was rapidly removed from a living torpedo, and prepared frogs were placed on the organ. On passing a knife into the organ, and dividing the smallest nervous filaments, the frogs leaped up, sometimes one, sometimes the other, according to the point of the electrical organ, which was cut. I had never before (says the author) seen in so perfect a manner the localised action of nervous filaments, nor had I ever witnessed so clearly the curious action of the electrical lobe of the brain. I received six torpedos, which were brought to me in a state of apparent inanition; the most active irritants failed to produce a discharge, for the animals seemed to have been destroyed by the cold. I exposed the brain, and on irritating the fourth lobe I obtained very powerful discharges. I cut up the electrical organ of a live torpedo in all directions, and applied the galvanometer to different points; the direction of the electrical current was invariably from the points nearest the back, towards the lower part of the belly. It is impossible to admit any analogy between the organ and piles, batteries, &c.—*Ibid, from Transactions of the Academy of Sciences.*

---

### STAMMERING.

At a meeting of the Medico-Chirurgical Society of Edinburg, Jan. 4, 1843, Dr. Abercrombie communicated "Observations on the Impediment of Speech, commonly called Stammering." When Dr. Abercrombie's attention was first directed to the subject, the following facts attracted his notice :—

I. He observed that stammerers never stammer in singing.

II. The individual on whom his first observations were made, did not stammer when he was obliged to speak in a louder tone of voice than usual, as when conversing in the midst of a noisy crowd, or in a carriage on a rough road.

III. The precentor of a church came under his notice, who stammered in common conversation, but showed no hesitation when reading out the line, as it is called, which was done in a peculiar high-pitched tone of voice, such as is usually employed by precentors for that purpose.

IV. He found that stammerers have no difficulty in performing any of those movements of the lips and the tongue, by means of which the consonant sounds are produced, when they are directed to make these movements simply—that is, without any reference to speech.

V. He observed that in some stammerers the difficulty is not confined to the consonant sounds in which the peculiar action of the organs of speech is more directly exerted, but extends to other sounds, in which these organs are little, if at all, concerned, such as the simple aspirated *h*, as in the words *happiness*, *holiness*, &c. In one individual, indeed, who was treated successfully, he found that he often hesitated at such words as these, long after he had overcome every difficulty respecting the consonants.

By such facts as these he was led to conjecture that the affection does not depend upon any defect in the organs of speech, properly so called; but is rather connected with a deficiency in the management of the voice, and he thought it would be found that, when a stammerer gets into that peculiar state of hesitation which is familiar to every one who has witnessed it, he is endeavouring to speak when he has no voice—that is, when the lungs have become emptied of air, or nearly so.

According to these views, the principles on which the cure of the affection may be accomplished appeared to be the following.—In actually accomplishing a cure, everything depends upon the perseverance of the patient himself after the principles have been explained to him.

I. To direct the attention of the individual to the three distinct parts of which the function of speech consists—viz.

1. A full and continuous current of air proceeding outwards from the lungs.

2. The formation of this into inarticulate sound, or voice, by the action of the larynx.

3. The formation of this into articulate sound, or speech, chiefly by certain movements of the lips and the tongue.

He soon perceives that he has no difficulty in performing any of these actions when they are thus made separate objects of attention; and in this manner he is led to understand that his affection does not depend upon any defect in any of the organs of speech, or a difficulty of performing any of the processes of which the function consists; but in a certain want of harmony among these processes which has grown into a habit. He is easily made to perceive, for example, that he has no difficulty in performing that motion of the lips by which is formed the sound of the letter *b*, then why should we have any difficulty in saying *bee*, *boy*, *bell*, &c. When the formation of each letter is thus made a separate object of attention, or a distinct voluntary act, it is remarkable to observe how the difficulty seems to vanish; and, by continued attention in this manner, the habit is gradually broken, in as far as concerns this part of the process.

II. The second and principal part of the treatment is, to exercise the individual in the habit of never attempting to speak without having A FULL AND STRONG CURRENT OF VOICE. He may be made sensible of the effect of this by making him read in a strong, loud tone of voice, as if he were calling to a person at a distance, or in a tone resembling singing or chanting, or in the peculiar tone of a precentor, in reading out the line, which has been already referred to. When he has thus been made to understand the principles on which the removal of the affection is to be conducted, the farther treatment consists in a course of exercises calculated to give him a full command of his voice, and so to correct the habit which he has acquired, of speaking, or attempting to speak, without sufficient voice. For this purpose he should be made to read aloud several times a-day, from an author whose style is somewhat declamatory. In doing so, he should be made to read in a high-pitched tone, and to stop frequently and take a full breath, so as to have the voice thrown out with a force beyond what is required for ordinary reading or ordinary conversation. With this view it is necessary to make him stop and take a full inspiration much more frequently than would be required by another person; for it is in this part of the process that we are to trace, in a great measure the bad habit he has acquired, and the opposite habit which he is required to cultivate. In particular, when he feels the tendency to hesitate at a word, he is to be taught to stop instantly, take a full breath, and then try it again. He will be immediately sensible of the effect; and a succession of voluntary efforts of this kind will be gradually formed into a habit, calculated to correct the injurious habit, in which, I believe, we are chiefly to trace the pathology of stammering.

In a note appended to this paper, Dr. Abercrombie remarked that, since his observations were written, he had found that the same principle respecting the influence of respiration in this affection had been pointed out by Dr. M'Cormack, of Belfast, in a small volume published in 1828.—*Edin. Monthly Journ.*

---

### REMARKS ON THE CALCULI IN ST. GEORGE'S HOSPITAL.

BY DR. BENCE JONES.

The number of specimens submitted to examination was 233. The author's object, from the analysis of these calculi, is to arrive at conclusions with regard to the comparative frequency of different states of the urine in calculous complaints, and thus to obtain practical hints as to the efficacy of remedies intended to alter the secretion, or act upon the stone in the bladder. He presents several tables; and taking 450 states of the urine, inferred from the composition of the calculi, finds that, in 139, it was alkaline, and in 311 acid, to test paper. Omitting from the latter list 59 specimens of oxalate of lime, 252 cases of the uric acid diathesis remain; and in 117 of these no free acid was passed, from which the author concludes that alkalies would have been of no benefit to them, so far as neutralising acidity of the urine was concerned. Taking the cases in which the alkaline concretions prevailed, he infers that in 52 the calculus might have been lessened by the injection of dilute acids; and in 12 the whole calculus might have been removed; while in others to which he refers, disintegration might have been effected. He concludes by describing a calculus in Mr. Cæsar Hawkins' possession, the nucleus of which consists of cystine, and which, from the history, appears to have been formed when the patient was two years and a half old.—*Trans. Royal Med. and Chirurg. Society, from Prov. Journ.*

### URETHROPLASTY.

At the Academy of Medicine, Paris, March 7, 1843, M. Ricord presented a patient on whom he had recently operated for loss of the urethra. The canal had been completely detroyed in its spongy portion by phagedenic chancre; the greater portion of the skin of the penis had also been destroyed by the same sore, and two-thirds of the circumference of the corpora cavernosa as well as the sulcus, indicating the original seat of the urethra, were covered by a thin cicatrix; the urine escaped through an opening in a fold of integument over the scrotum. To remedy this state of things, M. Ricord endeavoured to form a new urethra between the corpora cavernosa and the tissue of the cicatrix. For this purpose he passed a peculiar shaped trochar into the meatus urinarius, and pushed it backwards in the direction of the original urethra and above the cicatrix, until it met a gorget, which had been passed a few lines into and beyond the orifice through which the urine flowed. A silver canula was now introduced through the artificial canal, and allowed to remain in for two hours, when the urine passed freely through the new channel. The penis was enveloped in lint moistened with cold water; there was little tumefaction or inflammation. On the fifth day the canula was replaced by an elastic bougie, and since the 27th of January (the day on which the operation was performed) the size of the bougies has been gradually increased, so as to give a proper diameter to the new canal.—*Prov. Med. Journ.*

### PILULÆ HYDRARG. CUM FERRO.

Dr. G. F. Collier recommends a compound of the sesquioxide of iron and mercury, as preferable to the ordinary blue pill of the pharmacopœia. Its advantages he states are as follows:—It is made in five minutes, the ordinary blue pill requiring a week (in reality, many hours, not days.) The globules are not visible, even by the microscope; it is uniform in its appearance and effects; it makes a smoother pill, retaining its form more permanently; it salivates in a few days in the usual doses, the presence of the iron preventing the wear and tear of the frame under the effects of the mercury, and the powers of life are not so much (scarcely at all) prostrated under its use; it is, consequently, peculiarly eligible for the strumous, irritable, and anemic constitutions. It is prepared by rubbing together one part of the sesquioxide of iron, two of mercury, and three of rose confection, until the mercurial globules cannot be distinguished.—*London Lancet.*

### VALUE OF CARBONIC ACID WITH QUININE IN INTERMITTENT FEVERS.

A physician in a marshy district in the south of France having frequently met with cases of endemic intermittent fevers in which, owing to a spasmodic state of the stomach, the sulphate of quinine becomes inert, has found means to restore its active powers by associating it with carbonic-acid gas. The following preparations are those he recommends with this view:—℞. Tartaric Acid, 2½ drachms; Sulph. of Quinine, 1½ grains; rub well together, and add Bicarb. of Soda, 18 grains, and Powdered Sugar ½ drachm. To be taken while effervescing, in half a glass of water. ℞. Sulph. of Quinine, 12 grains; Tartaric Acid, 1 drachm; Bicarb. of Soda, 1¼ drachm, and Powd. Sugar, 1 oz., to be dissolved in a quart of water. Mix first the sugar and water, then the quinine with the tartaric acid; and lastly, the bicarb. of soda; seal hermetically to prevent escape of gas. Dose, a wine-glassful every two hours.—*London Lancet.*

### REVIVAL OF THE ARTS AMONG THE GREEKS.

Dr. Archigenes, a Greek surgeon, of Epibates, in Thrace (Roumelia,) has recently presented to the French Academie de Medicine an instrument which he has invented for operating upon vesico-vaginal fistula. This instrument, which is manufactured by M. Charriere, of Paris, is stated by its originator to preserve all the advantages, avoiding the inconveniences of others for the same purpose, invented by Depierris, Forrestier, and other French surgeons. The short notice we have received of its peculiarities would render its description here meagre or unintelligible.—*Ibid.*

### CELLULAR CYSTICERCUS UNDER THE CONJUNCTIVA.

M. Florent Cunier was called to see a patient who had a small vesicular swelling on the conjunctiva of the right eye, near the cornea, the result of an attack of inflammation following a blow from a butterfly, which had struck against the eye, and left one of its feet in the folds of the membrane. At the time M. Cunier saw it, it was about as large as a pea, and had several enlarged and varicose vessels feeding it; it was not painful, but impeded vision by its encroaching on the cornea. M. Cunier first punctured the vesicle, and the next day dissected it away with a pair of curved scissors. On examining the swelling after it had been withdrawn from the water into which it had been plunged, it was found to represent a transparent, vesicular body, with a swollen extremity, like a caoutchouc bag. When examined under the microscope, it was recognised as a cellular cysticerca, analogous to those that have been already observed in the same part by Baumés, Hœring, and Estling. The four suckers and the double circle of hooks were perfectly distinct. After having separated the worm from the remaining portion of cyst, and allowed it to be in water for two hours, it was found to have folded itself on itself, and looked like a piece of crystalline that had been in water for several days; the oblong spot, spoken of by M. Baumés as consisting of the retracted head and neck, were very distinct.—*Prov. Med. Jour., from Annales d'Oculistique.*

### ADULTERATION OF MILK.

M. Donne presented an instrument which he calls lactoscope, and which indicates the quantity of cream contained in any given specimen of milk. The principle of this instrument depends on a property of milk. It is well known that the dull white color of milk is occasioned by the number of rich globules which it contains; and the greater the number of these globules, the richer is the milk in cream. Now, as the opacity of milk depends on the quantity of globules, the measure of this opacity will give the measure of the richness of the milk. The opacity, however, cannot be appreciated in a fluid; only thin layers will serve for the purpose, and this is effected by the lactoscope. The instrument is composed of two parallel plates of looking-glass, which are brought together or removed from each other as required; the milk is placed between the plates, and the flame of a candle serves to measure the opacity, which is measured off on a graduated circle.—*Trans. Academy of Sciences, Paris, from Prov. Med. Jour.*

